# Comparative transcriptomic and metabolic analysis reveals the effect of melatonin on delaying anthracnose incidence upon postharvest banana fruit peel

**DOI:** 10.1186/s12870-019-1855-2

**Published:** 2019-07-01

**Authors:** Taotao Li, Qixian Wu, Hong Zhu, Yijie Zhou, Yueming Jiang, Huijun Gao, Ze Yun

**Affiliations:** 1 Guangdong Provincial Key Laboratory of Applied Botany, South China Botanical Garden, Chinese Academy of Sciences, Guangzhou, China; 2 Institute of Fruit Tree Research, Guangdong Academy of Agricultural Sciences, Guangzhou, China

**Keywords:** Anthracnose, Banana fruit, Cell wall, Melatonin; senescence, Volatile compounds

## Abstract

**Background:**

Banana anthracnose, caused by *Colletotrichum musae*, is one of the most severe postharvest diseases in banana. Melatonin is widely known for its role in enhancing plant stress tolerance. However, little is known about the control of melatonin on anthracnose in postharvest banana fruit.

**Results:**

In this study, exogenous melatonin treatment could significantly reduce the incidence of anthracnose in ripe yellow banana fruit and delay fruit senescence. However, melatonin treatment did not affect the growth of *Colletotrichum musae* in vitro. Transcriptomic analysis of banana peel showed that 339 genes were up-regulated and 241 were down-regulated in the peel after melatonin treatment, compared with the control. Based on GO terms and KEGG pathway, these up-regulated genes were mainly categorized into signal transduction, cell wall formation, secondary metabolism, volatile compounds synthesis and response to stress, which might be related to the anti-anthracnose of banana fruit induced by melatonin treatment. This view was also supported by the increase of volatile compounds, cell wall components and IAA content in the melatonin-treated fruit peel via the metabolomic analysis. After melatonin treatment, auxin, ethylene and mitogen-activated protein kinase (MAPK) signaling pathways were enhanced, which might be involved in the enhanced fruit resistance by regulating physiological characteristics, disease-resistant proteins and metabolites.

**Conclusions:**

Our results provide a better understanding of the molecular processes in melatonin treatment delaying banana fruit senescence and anthracnose incidence.

**Electronic supplementary material:**

The online version of this article (10.1186/s12870-019-1855-2) contains supplementary material, which is available to authorized users.

## Background

Melatonin, known as N-acetyl-5-methoxytryptamine, is an animal hormone that is involved in the regulation of various physiological processes including sleep physiology, circadian rhythms, and sexual behaviors [1]. Besides the above mentioned functions, melatonin is also well known as a powerful free-radical scavenger and wide-spectrum antioxidant [2]. Since the first discovery of melatonin in plants [3], a widespread existence of melatonin in plant kingdom has been reported in a considerable variety of plant species, and melatonin plays an important role in many biological processes, such as seed germination [4], flower development [5], leaf senescence [6], fruit ripening [7], response to cold stress [8] and response to pathogen [9]. However, the effect of melatonin on banana senescence is not clear, especially on postharvest banana fruit after ripening.

Banana is a typical climacteric fruit that firstly goes through ripening after harvest, followed by senescence, which causes the fruit deterioration and shorten its shelf-life. In this process, fruit becomes vulnerable to pathogenic microorganism and saprophytes’ attack. Generally, the degree of fruit disease gradually aggravates with the deepening of fruit senescence. Banana anthracnose caused by *Colletotrichum musae* is one of the most serious postharvest diseases in banana worldwide [10]. In our preliminary experiment, melatonin treatment significantly reduced the anthracnose incidence of banana fruit (from unpublished data). In the view of safety and important biological effects, melatonin can serve as a good candidate for the control of anthracnose. Melatonin is a signal exciter in plants that significantly inhibits leaf senescence retardation [6] and pathogen attack [9] though triggering auxin, ethylene and salicylic acid signal pathways. Melatonin can also enhance the antioxidant capabilities of plants, and remove reactive oxygen species by enhancing ascorbic acid peroxidase [11]. A further study showed that exogenous application of melatonin improved banana resistance to *Fusarium* wilt through induced MaHSP90s [12]. However, little is known about the mechanism of melatonin in regulating the banana anthracnose.

Recently, ‘omics’ technologies including transcriptomics and metabolomics have provided a global view of changes in the abundance of transcripts and metabolites in spatial, temporal, or conditional manners, enabling us to investigate the complexity of the postharvest fruit physiology. Due to the obvious advantage and sensitivity of RNA-seq, more researchers use deep RNA-sequencing combined with digital gene expression profile (DGE) analysis to rapidly identify and analyze the dynamic of fruit physiology [13]. While sequenced banana genome has provided a good reference for DGE analysis, the integrated analysis of transcriptomics and metabolomics research on banana has not been documented, and the role of melatonin in controlling banana anthracnose has not been reported.

In this study, to explore the regulatory mechanism of melatonin in delaying banana anthracnose, comparative transcriptomic and metabolomic analyses were performed to investigate and identify differentially regulated genes and metabolites after melatonin treatment. The results of our study are beneficial for the application of melatonin on postharvest quality improvement of banana fruit as well as other horticultural fruit.

## Results

### Physiological characteristics of harvested banana after melatonin treatment

**Fig. 1:**
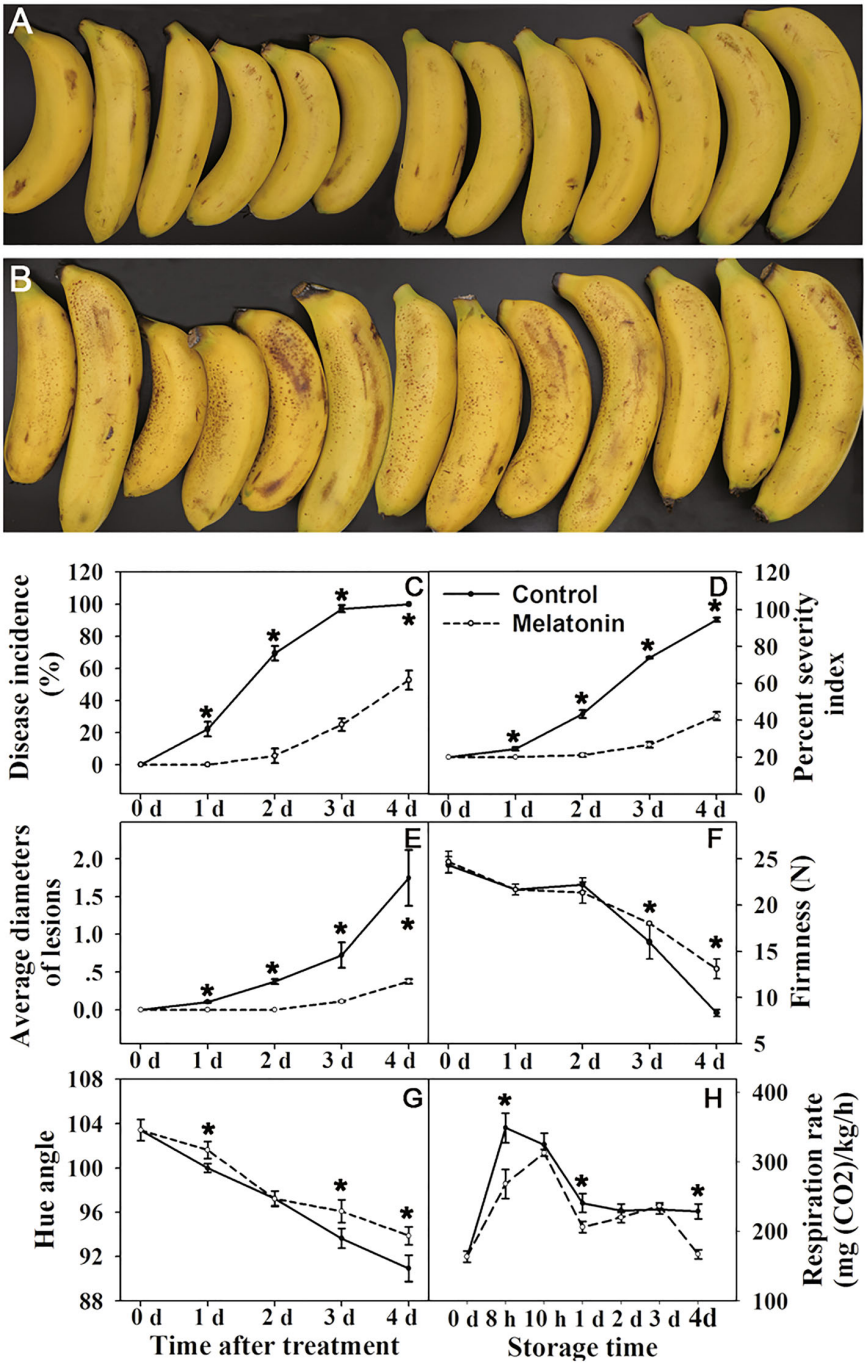
Effect of melatonin on incidence of banana anthracnose. **a** melatonin-treated banana fruit. **b** control banana fruit. **c** anthracnose incidence. **d** percentage of incidence area. **e** average diameter of anthracnose spot. **f** fruit firmness. **g** fruit color. **h** fruit respiration rate

To verify the effect of melatonin on inducing fruit resistance, yellow ripe banana fruit were soaked in 10 mM melatonin for 3 min. It was shown that melatonin treatment significantly reduced the anthracnose incidence and fruit senescence (Fig. 1a and b). On Day 4, the disease incidence of the control was 100%, while the incidence of fruit treated with melatonin was 50% (Fig. 1c). The percent severity index of the control was nearly to 100%, while that of the melatonin treated fruit was 40% (Fig. 1d). The disease spot diameter was more than 1.5 mm in the control compared with 0.4 mm in the melatonin-treated fruit (Fig. 1e). In addition, melatonin treatment significantly inhibited the decrease of fruit firmness and hue angle value (Fig. 1f and g), and reduced the fruit respiration rate (Fig. 1h). In all, the melatonin treatment significantly delayed banana fruit senescence and significantly reduced the anthracnose incidence during the shelf-life of banana fruit.

### Physiological characteristics of *Colletotrichum musae* after melatonin treatment

**Fig. 2:**
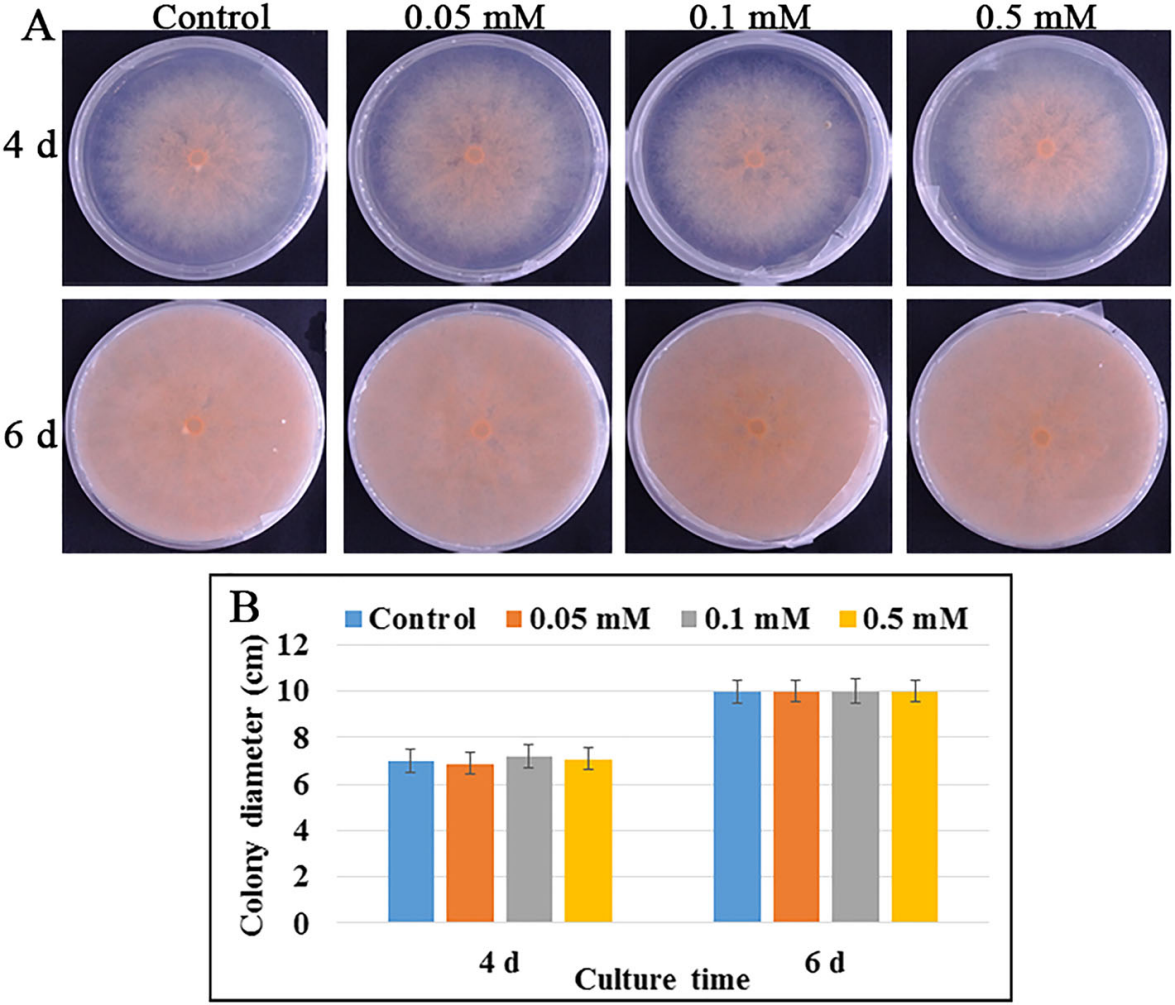
**a** Colletotrichum musae was isolated and purified from banana fruit peel using PDA medium. Colletotrichum musae was cultured on PDA medium containing 0 (control), 0.05, 0.1 and 0.5 mM melatonin, respectively. **b** The colony diameter was observed and measured at 4 d and 6 d of culture

There might be two ways in melatonin-reduced anthracnose incidence of banana fruit; one is inducing fruit disease resistance, and the other is killing *Colletotrichum musae* on the fruit surface. To verify the toxicity of melatonin to *Colletotrichum musae*, *Colletotrichum musae* was isolated and purified from banana fruit, and cultured on PDA medium with and without melatonin. Result showed that the growth of pathogenic fungus was not significantly affected by the treatment of three concentrations of melatonin during the colony culture period (Fig. 2a and b), suggesting that melatonin treatment may reduce banana fruit anthracnose incidence merely by inducing fruit disease resistance.

### Transcriptomic analysis of banana peel after melatonin treatment

To investigate the gene expression profiling of banana peel after melatonin treatment, Agilent 2100 Bioanaylzer and ABI StepOnePlus Real-Time PCR System are used to qualify and quantify of the sample library. More than 12,000,000 clean reads were obtained from each replicate, and total mapped reads were more than 9,000,000 in each replicate (Additional file 6: Table S1). After gene expression and differentially expressed genes analysis, 339 genes were up-regulated and 241 genes were down-regulated after melatonin treatment.

**Fig. 3:**
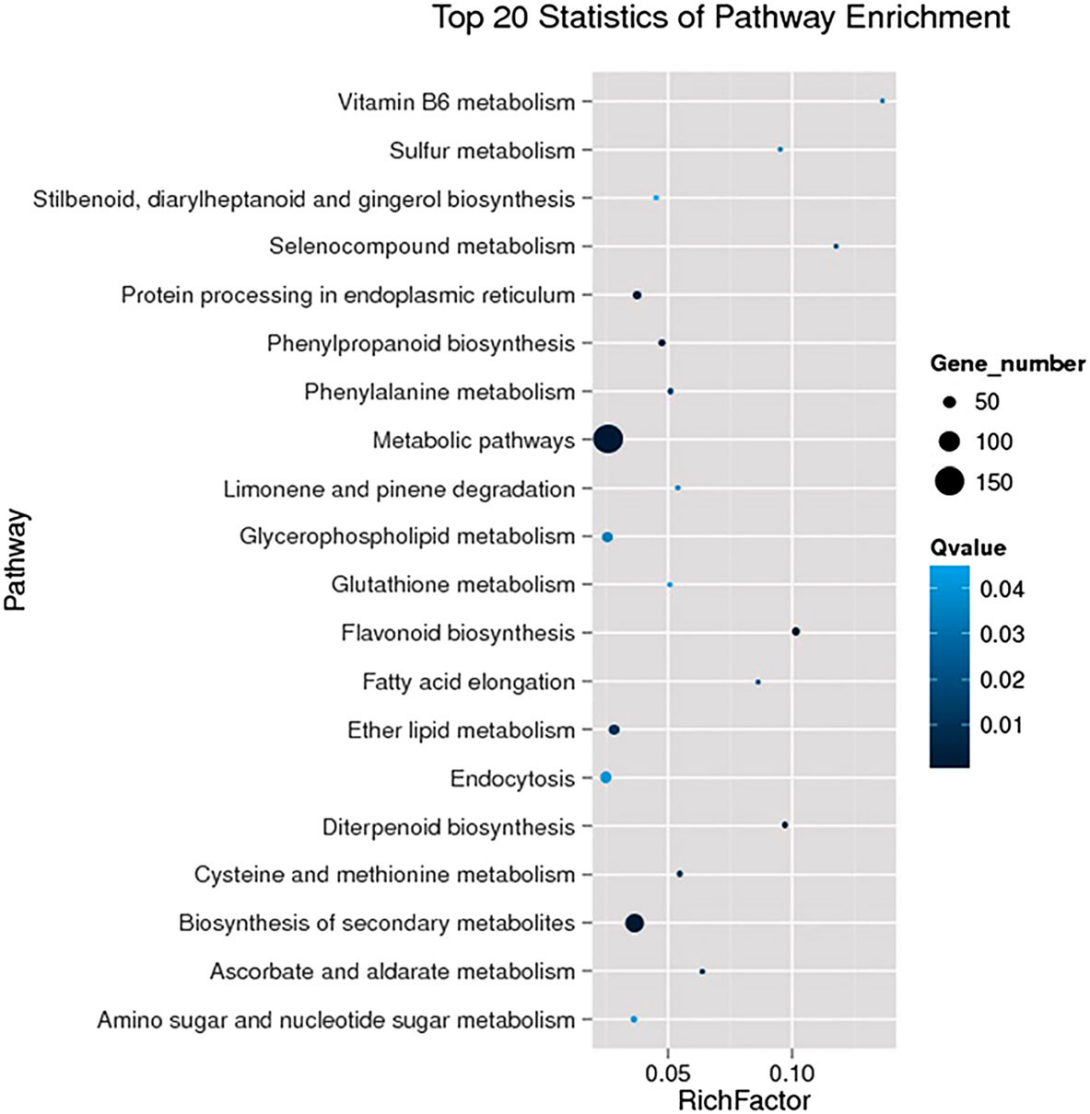
Top 20 statistics of pathway enrichment for differentially expressed genes after KEGG pathway analysis

To obtain more information about the differentially expressed genes, gene function clustering analysis was conducted using Blast2GO according to biological processes, molecular function and cellular components. For biological processes, the genes were distributed among 20 categories (Additional file 1: Figure S1), the largest one being ‘metabolic process’, followed by ‘cellular process’, ‘response to stimulus’ and ‘single-organism process’. KEGG pathway analysis classified the 580 differentially expressed genes into 88 categories, of which the top 20 significant enrichment pathways were shown in Fig. 3. ‘Metabolic pathways’ and ‘biosynthesis of secondary metabolites’ were the largest groups with highest significance, suggesting that both categories of genes have pivotal roles in the melatonin-induced banana disease resistance.

**Fig. 4:**
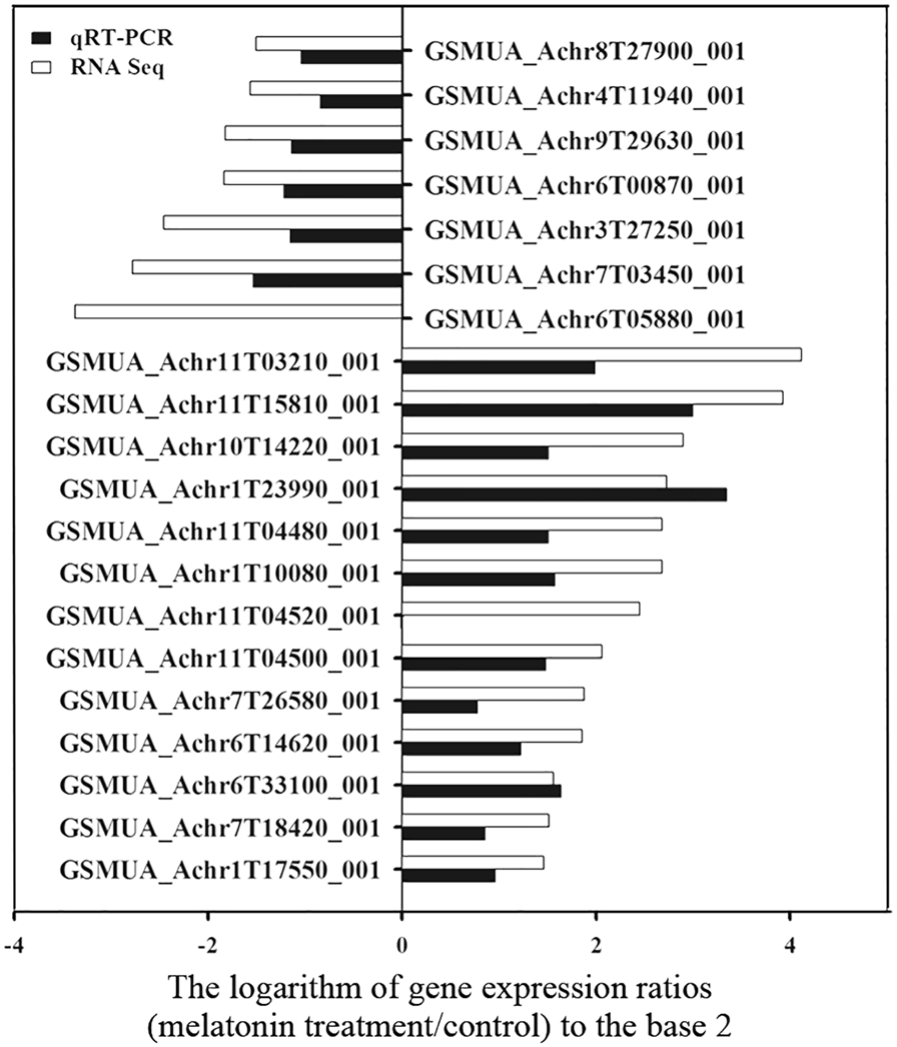
Verification of twenty selected differentially expressed genes from RNA-seq using qRT-PCR

To verify the validity of RNA-seq data, we used real-time quantitative PCR (q-PCR) to analyze the expression patterns of twenty genes, mainly involved signal transduction, transcription factors, response to stress, cell wall, and other metabolic pathways. Result showed that the expression patterns of those genes were consistent between q-PCR and RNA-seq data, and the fold changes of most genes were greater in RNA-seq data than in q-PCR data (Fig. 4).

### Differentially expressed genes related to signal transduction and transcription factors after melatonin treatment

A total of 74 genes involved in signal transduction were differentially expressed, among which 15 of them were down-regulated and 59 of them were up-regulated after melatonin treatment (Additional file 9: Excel S1). According to ClueGo analysis by Cytoscape (version 3.3.0), these genes were mainly clustered into receptor signaling pathway and cell wall organization (Additional file 2: Figure S2). Nineteen differentially expressed genes were annotated into transcription factors; 14 of them were up-regulated by melatonin treatment, including *WRKY, MYB, ERF, ARF and bHLH3* (Additional file 9: Excel S1). After BingGO analysis, the up-regulated transcription factors were mainly involved in hormone signaling, such as auxin signaling, ethylene signaling (Additional file 3: Figure S3). Those genes, including receptor signaling pathway, cell wall organization, auxin signaling and ethylene signaling, might play an important role in disease resistance of banana fruit induced by melatonin.

### Differentially expressed genes related to stress response

After melatonin treatment, 145 genes related to stress response were differently expressed. Ninety-one of them were up-regulated and 54 were down-regulated after melatonin treatment (Additional file 9: Excel S1). The analysis of homologous sequence showed that 580 differentially expressed banana genes could be matched to 334 *Arabidopsis* genes. MapMan (3.6.0RC1) analysis was performed by using *Arabidopsis* gene ID. The stress mainly included biotic stress and abiotic stress. Mapman analysis showed that 125 genes were involved in biotic stress while 13 genes involved in abiotic stress (Additional file 4: Figure S4), indicating that melatonin treatment could enhance the resistance of banana peel to biotic stress. In ‘response to biotic stress’, melatonin treatment induced genes were mainly involved in auxin, ethylene, MAPK, peroxidases, cell wall, proteolysis, heat shock protein and secondary metabolites (Additional file 4: Figure S4). Due to the absence of homologous alignment of some banana gene sequences to *Arabidopsis* genes, the number of genes in Mapman analysis was less than the actual number, and the number of genes listed in the subsequent was based on the KEGG pathway analysis.

In terms of signal transduction, 26 genes were up-regulated after melatonin treatment, including eight including eight genes related to ethylene signaling, four genes related to auxin signaling, ten genes related to ROS and four genes related to MAPK signaling (Additional file 9: Excel S1). Moreover, eight receptor proteins were up-regulated by melatonin treatment (Additional file 9: Excel S1). Curiously, only a few of pathogenesis-related (PR) proteins were up-regulated significantly after melatonin treatment, including four *peroxidases* and two *lipid-transfer protein*.

### Differentially expressed genes related to metabolism after melatonin treatment

**Fig. 5:**
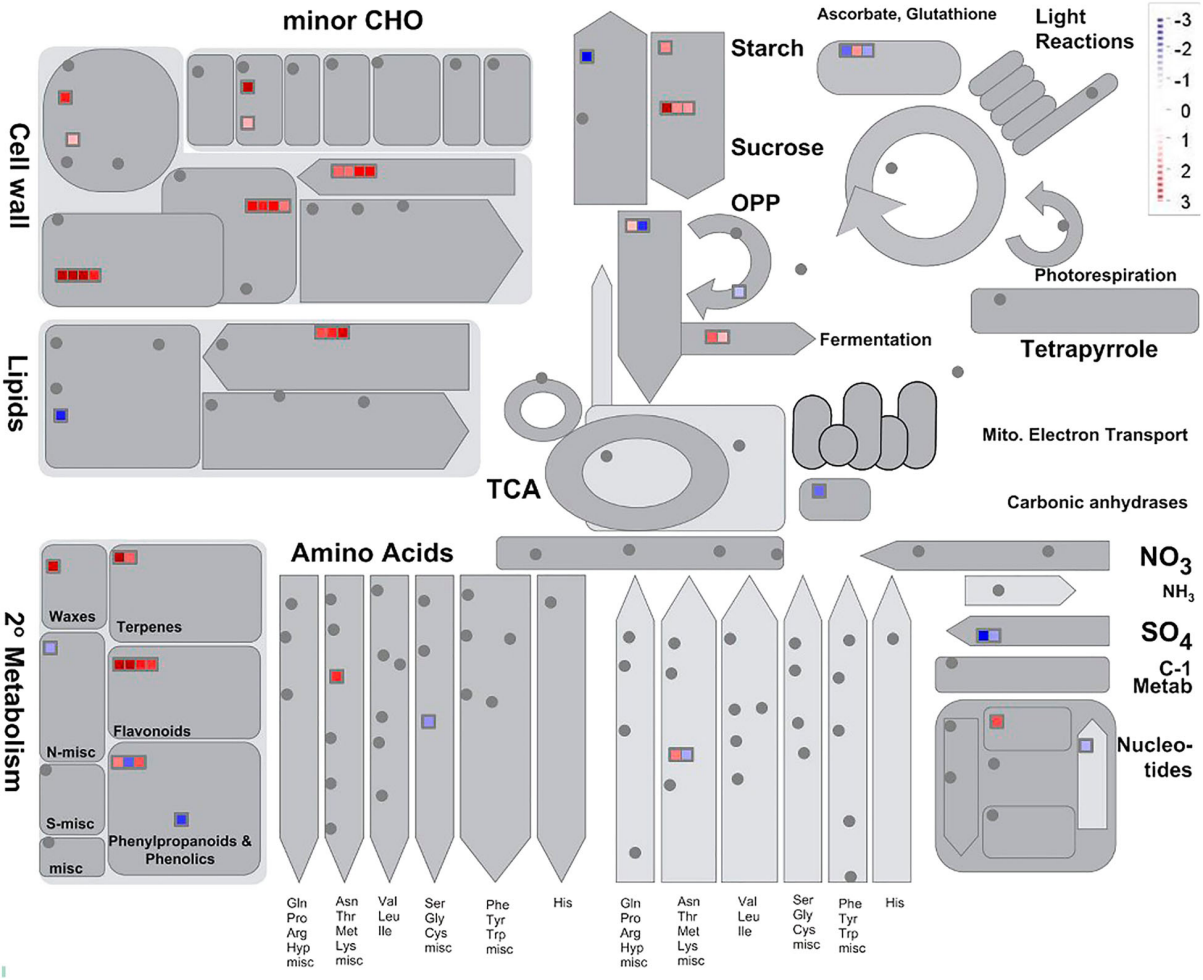
Schematic of the metabolism pathway using the MapMan visualization platform. The analysis of homologous sequence showed that 580 differentially expressed banana genes could be matched to 334 *Arabidopsis* genes. MapMan analysis was performed by using *Arabidopsis* gene ID. The logarithm of gene expression ratios (melatonin treatment/control) base 2 were used in MapMan analysis. The red or green squares indicate the up or down-regulated genes involved in corresponding metabolism

In this study, 155 genes related to metabolic process were differently expressed after melatonin treatment. MapMan analysis showed that melatonin-induced metabolic processes are mainly involved in the synthesis of cell wall, lipids, flavonoids, waxes and terpenes, and starch degradation (Fig. 5). After melatonin treatment, 22 genes involved in the cell wall metabolism were differentially expressed. Only four of them showed down-regulation in the melatonin-treated banana peel, and 18 up-regulated genes were predicted to be involved in the biosynthesis of cell wall, such as probable xyloglucan endotransglucosylase/hydrolase proteins and cellulose synthase (Additional file 9: Excel S1). There were 32 differentially expressed genes related to lipid metabolism, 22 of which were up-regulated after melatonin treatment, mainly involving sphingolipid and lipid biosynthetic process, lipid oxidation and membrane lipid biosynthetic process (Additional file 9: Excel S1). For waxes synthesis, five *3-ketoacyl-CoA synthases* were up-regulated in melatonin-treated banana peel (Additional file 9: Excel S1). For flavonoid biosynthesis, 14 genes were up-regulated by melatonin treatment (Additional file 9: Excel S1), including six *S-norcoclaurine synthases* (*NCS1*), two *chalcone synthases*, *rhamnose biosynthetic enzyme* and other genes (Additional file 9: Excel S1).

### Identification and analysis of volatile compounds in banana peel

**Fig. 6:**
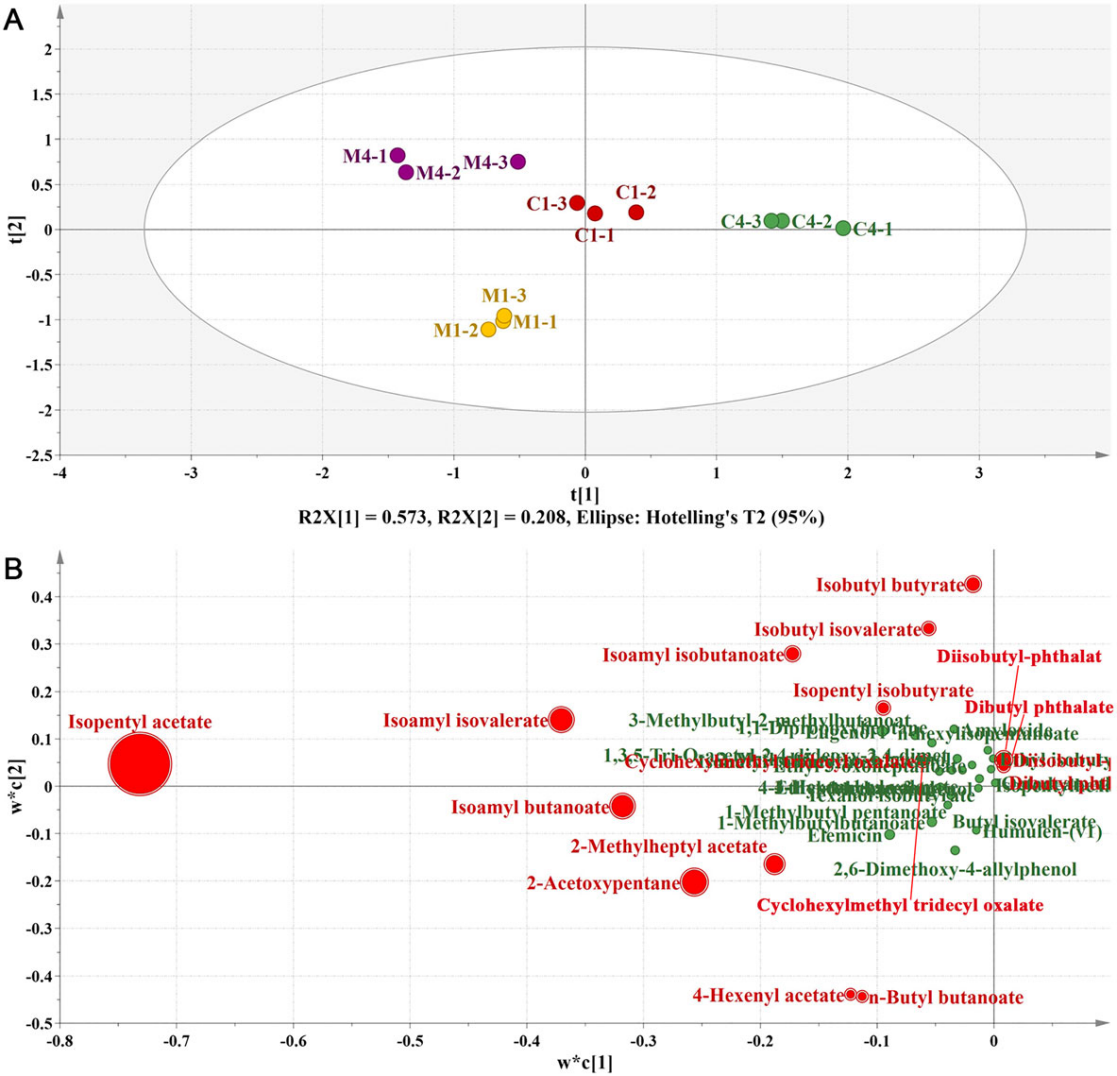
PLS-DA and OPLS-DA of volatile compounds in banana peel. A total of thirty-seven kinds of volatile compounds were performed PLS-DA and OPLS-DA. The result of OPLS-DA and ANOVA showed fourteen kinds of volatile compounds were important (predictive VIP > 1 and *p* < 0.05). **a** score scatter plot of PLS-DA. M1 means melatonin-treated sample on Day 1, C1 means control sample on Day 1, followed by the replicate number. **b** loading scatter plot of PLS-DA. The important compounds are in red color. The size of the circle represents the relative content of the compounds in control peel on Day 1

In order to investigate the aroma change after melatonin treatment, the content of volatile compounds in banana peel were analyzed using GC-MS. A total of 44 volatile compounds were identified in banana peel and the major volatile compounds were shown in Additional file 5: Figure S5. Among these compounds, the contents of 37 changed significantly (*p* < 0.05) after melatonin treatment, mainly including esters and phenolic substances (Additional file 7: Table S2). All these volatile compounds with significantly altered accumulation were used for projection to latent structures discriminant analysis (PLS-DA) by using SIMCA software (Version 15.0, Umetrics, Umea, Sweden). Result showed that R2X [1] and R2X [2] accounted for 0.781 of the variance in the dataset (Fig. 6a). Obvious separation was observed between control and melatonin-treated sample groups (Fig. 6a). Most of the volatile compounds loaded in PLS-DA were distributed in the direction of melatonin-treated pericarp samples (Fig. 6b), indicating that the contents of volatile compounds in banana peel changed significantly after melatonin treatment, which might play a vital role in melatonin-delayed banana fruit senescence. To further explore the important compounds in this study, orthogonal projection to latent structures discriminant analysis (OPLS-DA) was performed. A total of 14 volatile compounds, with predictive VIP > 1, were recognized as important compounds and shown in red color in Fig. 6b. These compounds were relatively high in banana peel.

During fruit senescence, the contents of 13 kinds of important volatile compounds decreased significantly, while only 4-hexenyl acetate increased. After melatonin treatment, the contents of seven important volatile compounds significantly increased on Day 1 compared to the control, and all volatile compounds were significantly induced on Day 4, including dibutyl phthalate, diisobutyl-phthalat, isobutyl butyrate, isobutyl isovalerate, cyclohexylmethyl tridecyl oxalate, isopentyl isobutyrate, isoamyl isobutanoate, 2-acetoxypentane, 2-methylheptyl acetate, isoamyl isovalerate, isoamyl butanoate, isopentyl acetate, n-butyl butanoate and 4-hexenyl acetate. Our results indicated that melatonin treatment was beneficial for maintaining the volatile compounds of banana peel and inhibiting fruit senescence.

### IAA content and cell wall components in banana peel after melatonin treatment

**Fig. 7:**
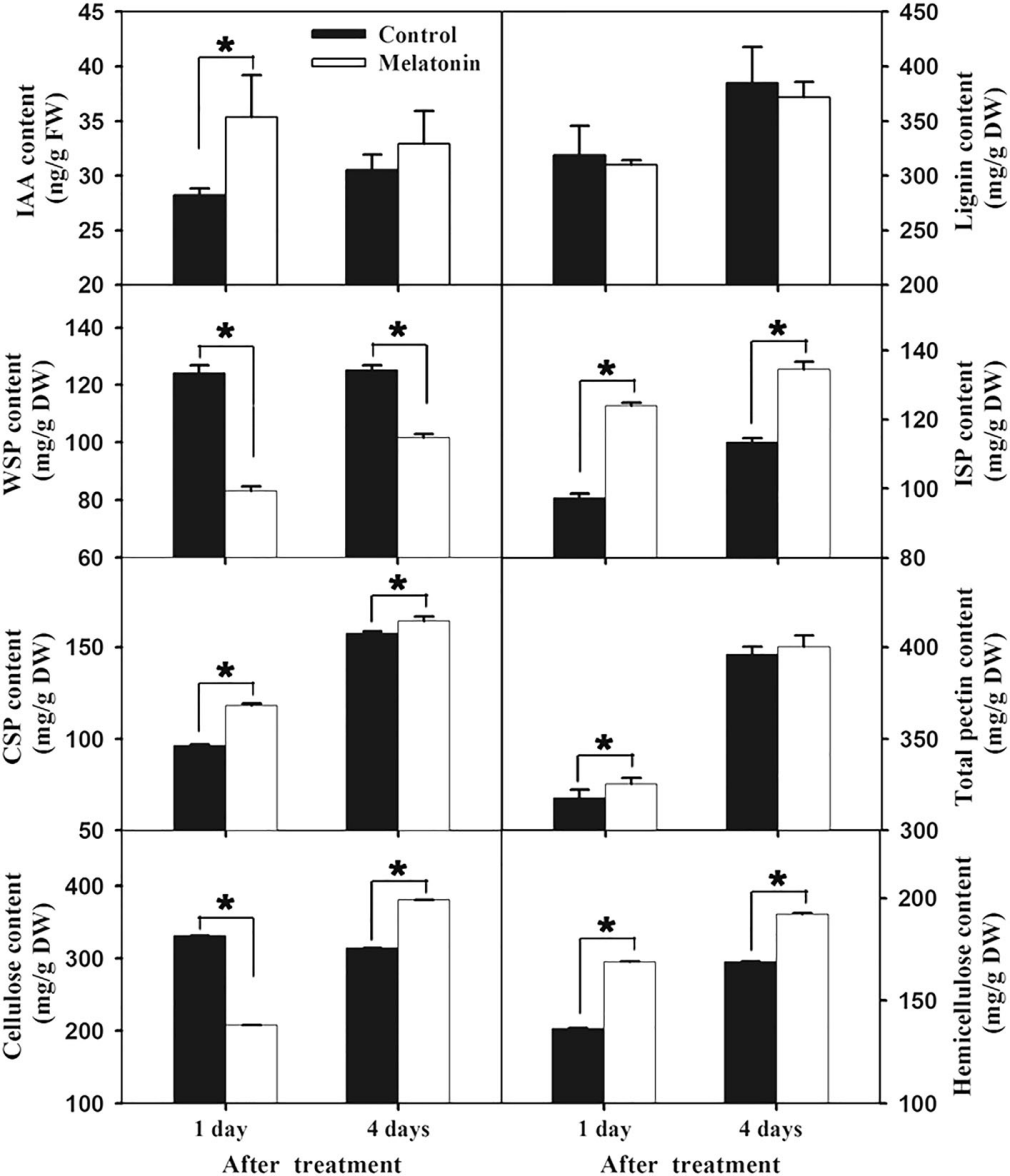
IAA and cell wall components contents after melatonin treatment. FW: fresh weight. DW: dry weight. WSP: water-soluble pectin. ISP: ionic-soluble pectin. CSP: covalent-soluble pectin

To verify the authenticity of auxin signal in melatonin treatment, IAA content was measured. It was shown that IAA level increased significantly on Day 1 after melatonin treatment, and then decreased (Fig. 7), suggesting that melatonin caused an instantaneous but not durable induction of IAA synthesis. The contents of cell wall components were also determined, including cellulose, hemicellulose, water-soluble pectin (WSP), ionic-soluble pectin (ISP), covalent-soluble pectin (CSP) and lignin. The contents of ISP, CSP, and hemicellulose significantly increased after melatonin treatment, only WSP content decreased significantly (Fig. 7). There seemed to be a transformation tendency from WSP to ISP and CSP. Strangely, the cellulose content was lower in melatonin-treated sample than in control on Day 1, but higher on Day 4 (Fig. 7).

### Coordinated changes of differentially expressed genes and fruit physiological characteristics of banana peel after melatonin treatment

**Fig. 8:**
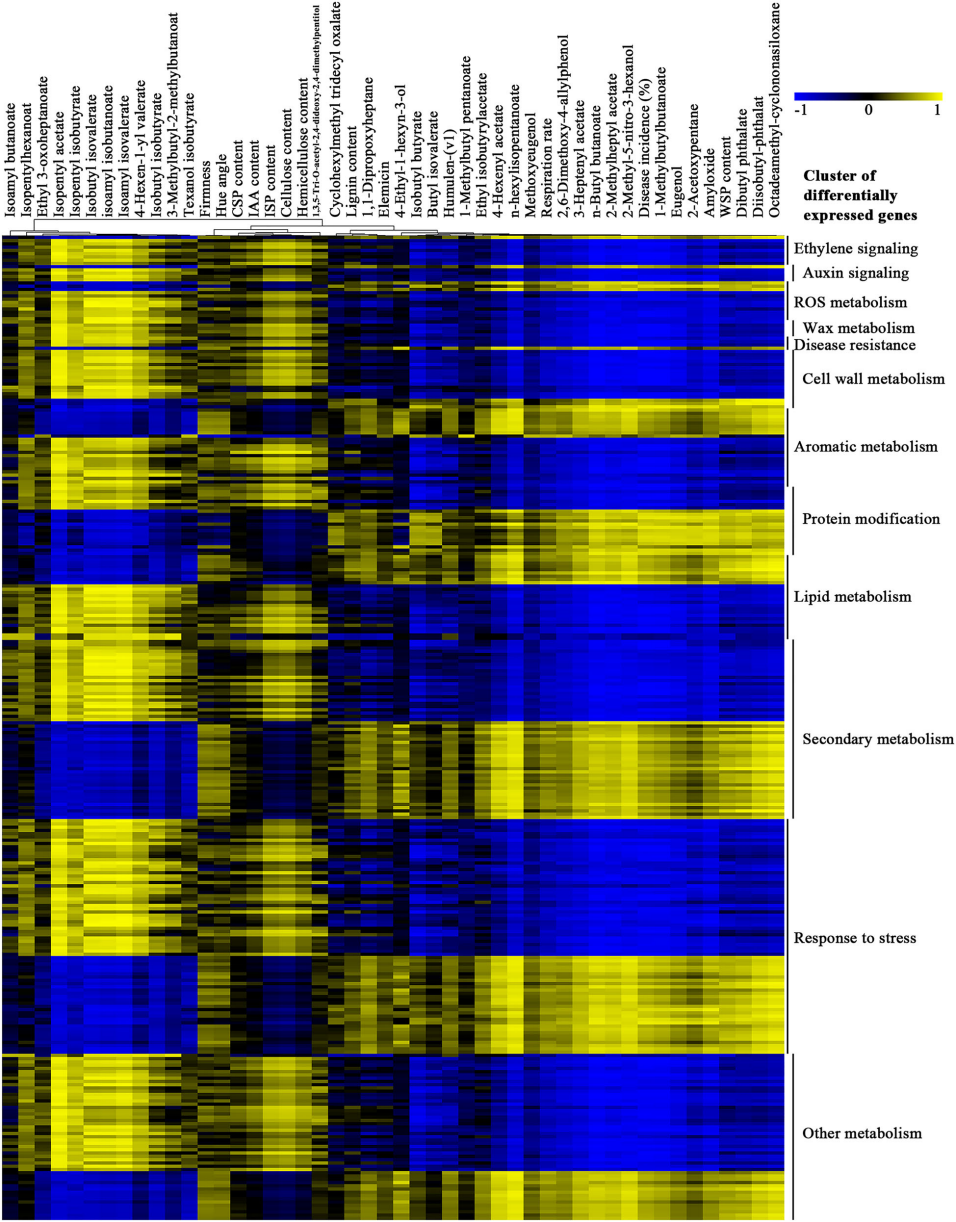
Heat map showing the correlation analysis between differentially expressed genes and fruit physiological characteristics of banana peel after melatonin treatment. A total of 302 significant (*p* < 0.05) expressed genes and 48 physiological characteristics were used for correlation matrix analysis. The correlation coefficient (positive or negative) is represented by virtual color as indicated in the color key

Correlation analysis was used to identify differentially expressed genes that were functionally related or co-regulated with fruit physiological characteristics of banana peel (Fig. 8). The coordinated shift in metabolites was evaluated by pairwise correlation analysis, and 302 significant (*p* < 0.05) expressed genes and 48 physiological characteristics were performed in this study. The results showed that most of ethylene and auxin signaling-related genes are positively correlated with fruit firmness, hue angle, IAA, ISP, CSP, cellulose, hemicellulose and most of volatile compounds with high levels, and the above physiological characteristics were positively correlated with most of genes related to ROS metabolism, wax metabolism, disease resistance and cell wall metabolism (Fig. 8). In contrast, only some genes in other clusters were positively correlated with the above physiological characteristics, including aromatic metabolism, protein modification, lipid metabolism, secondary metabolism, response to stress and other metabolism (Fig. 8). It is suggested that melatonin treatment can induce up-regulation of some genes related to a series of metabolic pathways in the banana peel, which results in a significant increase in the content of most substances, including cell walls, waxes, lipids and volatile components.

## Discussion

In the last two decades, the in vivo roles of melatonin have been documented in leaf senescence, plant development and stress responses [6, 7, 14], and remarkable advances have been made in the understanding the role of melatonin in delaying leaf senescence [6, 11]. For leaf, there is a process of macromolecule degeneration and nutrient recycling. However, fruit senescence is very different from leaf senescence. Fruit senescence is closely coupled with fruit ripening that makes fruit succulent and appealing to seed-dispersal vectors including humans, usually without a nutrient recycling process. Also, fruit senescence is a complex physiological and biochemical process, involving a series of physiological changes, such as carbohydrate and cell wall degradation, flavor reduction, and wax degradation.

### Melatonin treatment significantly delayed the anthracnose in banana fruit

Banana is a typical climacteric fruit and its shelf-life is short after ripening. The symptoms of anthracnose usually aggravate with the deepening of fruit senescence. Anthracnose, caused by *Colletotrichum musae* fungus, is the most important postharvest disease and seriously affects the quality and shelf-life of banana fruit [15]. Melatonin has been previously reported to improve fruit resistance against pathogen infection [14, 16]. In present study, melatonin treatment significantly delayed the occurrence of anthracnose of banana fruit, but had no effect on the fungus growth and development (Figs. 1 and 2). Meanwhile, the treatment significantly delayed fruit senescence, indicating that there might be some connections between fruit senescence and disease occurrence. So, comparative transcriptomic and metabolomic analysis of banana peel after melatonin treatment was performed. The discussion focused on the molecular mechanism of banana senescence and anthracnose delayed by melatonin.

### Melatonin treatment activated both ethylene and auxin signals in banana peel

Receptor-like kinases (RLKs) in plants are a large superfamily of proteins, which are involved in a series of plant responses, including development, growth, hormone perception and the response to pathogens [17]. In this study, some significantly up-regulated genes by melatonin were annotated as receptor protein kinase, such as *leucine-rich repeat receptor protein kinase*, *cysteine-rich receptor-like protein kinase 25* and *wall-associated receptor kinase 5*. Receptors and the signaling pathway of melatonin have also been demonstrated in animals [18]. However, the receptor-independent mechanism of melatonin in plants remained unclear. Although no high-affinity receptor for melatonin has been identified in plants to date, our results could provide a clue to investigate the receptor-mediated mechanism of melatonin.

**Fig. 9:**
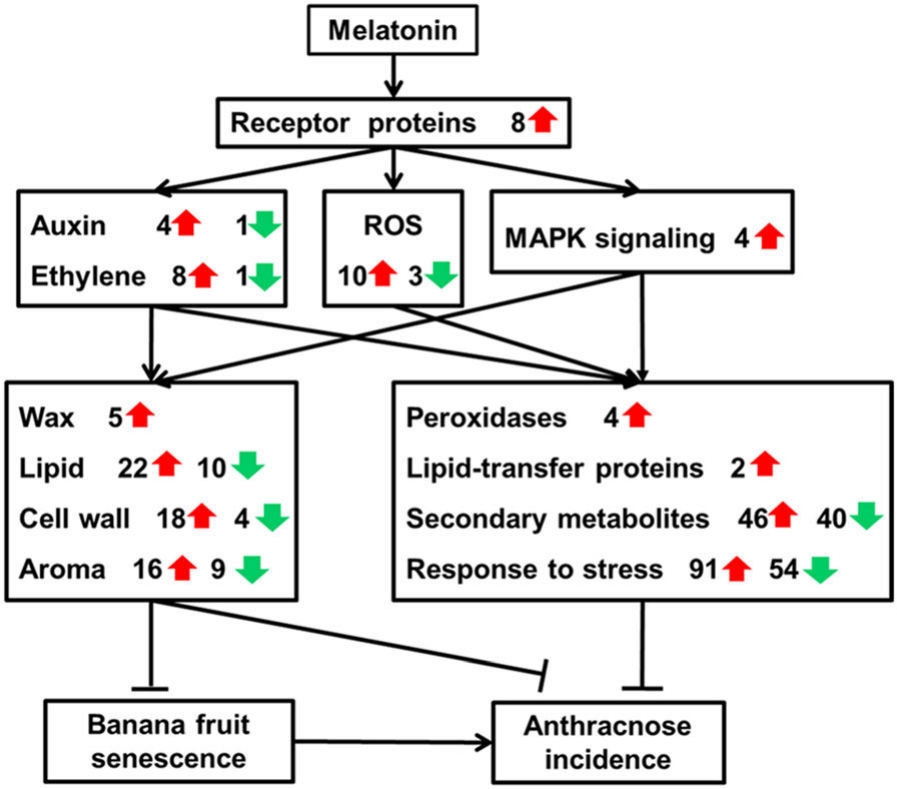
The principal pathways involving in the role of melatonin in delaying banana anthracnose incidence

Ethylene is important for fruit ripening and plant defense signaling [19]. In this study, the expression of ethylene biosynthesis genesand *ERFs* were significantly un-regulated after melatonin treatment (Additional file 9: Excel S1). The induced expression of *ERF*s and *ACO*s could regulate fruit softening, aroma release, and disease resistance enhancement [20]. Auxin signaling is well known for its role in plant development. In recent years, auxin related genes have been reported as involved in plant responses to pathogen infection [21]. There is crosstalk between auxin and ethylene signal, although detailed information was still lacking [22]. In this study, two genes encoding *indole-3-acetic acid-amido synthetase* and *auxin-induced protein* were induced in the melatonin-treated banana peel (Additional file 9: Excel S1). Correspondingly, the IAA content significantly increased after melatonin treatment (Figs. 7 and 8). In *Arabidopsis*, IAA was also reported to be a positive modulator of natural leaf senescence [6]. Taken together, both ethylene and auxin signals were activated in banana fruit after melatonin treatment (Figs. 8 and 9), revealing that melatonin might regulate banana senescence and disease resistance mainly though the activation of plant hormone signaling, such as ethylene and auxin.

### Melatonin treatment might induce banana fruit resistance though ROS-mediated signaling and MAPK signaling pathway

Reactive oxygen species (ROS) are reported to be involved with plant stress response [23]. In addition, respiratory burst oxidase homologue, as one of plant NADPH oxidase, is the major ROS source in plants. Our results showed that *respiratory burst oxidase homolog protein B* was significantly up-regulated after melatonin treatment, which might accelerate the response signaling in banana peel to resist pathogen infection (Fig. 9). Balance of ROS metabolism is critical for the normal life of plants. As ROS scavengers, one *catalase* and four *peroxidases* were up-regulated after melatonin treatment, which was consistent with previous findings in cassava storage roots [24]. The up-regulation of those genes increased the capability to scavenge ROS in banana fruit, and enhanced the wall-building process through oxidation of phenols, suberization and lignification [25]. Previous research also indicated that melatonin can induce the *peroxidase* expression to inhibit the senescence of apple leaf [11]. It suggested that melatonin treatment might induce banana fruit resistance though ROS-mediated signaling pathway (Fig. 9).

MAPKs signal pathway is also involved in plant stress response [26]. Pathogen-responsive MAPKs can be triggered in melatonin-mediated defense responses in *Arabidopsis* and tobacco, which may induce a series of defense-related genes via various phosphorylated transcription factors [14]. Moreover, MAPK signaling could interact with WRKY33 to regulate the expression of camalexin biosynthetic genes, which drives the metabolic flow to camalexin production in *Arabidopsis* [27]. In this study, *mitogen-activated protein kinase 5* was up-regulated after melatonin treatment. Meanwhile, several WRKY transcription factors including WRKY33 were significantly up-regulated (Additional file 9: Excel S1). Considering our present results and previous reports, we postulated that MAPK signaling might be involved in the melatonin-induced banana anthracnose resistance.

### Melatonin treatment induced cell wall and wax synthesis in banana peel

Plant cell wall plays an important role in response to pathogen infection and can effectively insulate microbial pathogens from the products of plant metabolism. Intact cell wall can effectively protect the plants from pathogen infection [28]. In this study, 18 cell wall-related genes were significantly up-regulated after melatonin treatment (Additional file 9: Excel S1). Most of them are positively correlated with cellulose, hemicellulose, pectin and fruit firmness in banana peel (Fig. 8). In addition, comparative analysis showed that the contents of pectin, cellulose and hemicellulose were higher in melatonin-treated samples compared to the control on Day 4 (Fig. 7). It suggests that melatonin treatment maintains higher integrity of the cell wall, which is benefit for delaying banana fruit senescence and protecting the fruit from pathogen infection.

Fruit secretes waxes on the surface or into the cuticles to prevent water loss, retard shrinkage and spoilage, and improve appearance [29]. The decay index also significantly decreased in artificially wax-treated fruit [30]. The epicuticular waxes of plants are mixtures of substituted long-chain aliphatic hydrocarbons. Very-long-chain fatty acids are essential precursors of cuticular waxes in plants, and 3-ketoacyl-CoA synthases are key enzymes in very-long-chain fatty acids biosynthesis [31]. In this study, five *3-ketoacyl-CoA synthases* showed higher expression in the melatonin-treated fruit than in the control. An increase of 10–34% total wax was observed in transgenic *Arabidopsis* leaves overexpressing *KCS20* and *KCS2/DAISY*, and a 15–20% decrease was detected in *3-ketoacyl-CoA synthase* mutant [31]. It indicated that melatonin treatment induced banana fruit wax synthesis in peel, which also played a vital role in reducing anthracnose incidence.

Cutin is an important component of cuticle, which play a vital role in plant response to pathogen infection [32]. Cutin biosynthesis is involved in lipid transfer proteins, which are responsible for the shuttling of phospholipids and other fatty acid groups between cell membranes [33]. They are also known to play important roles in the resistance to biotic and abiotic stress [34]. In this study, two *lipid transfer proteins* genes were identified with up-regulation in the melatonin-treated sample (Additional file 9: Excel S1). Meanwhile, the wax and cutin biosynthesis related genes (*MYB*) were also up-regulated after melatonin treatment (Additional file 9: Excel S1). In sum, we carefully concluded that melatonin treatment could increase *MYB* expression, enhance wax and cutin biosynthesis, and protect banana fruit from *Colletotrichum musae* infection.

### Melatonin treatment could induce volatile compounds and lipids metabolism

Volatile compounds are produced during fruit ripening and most of them also serve as ‘biological bribes’ for the attraction of animals and as protectants against pathogens [35]. Our study found that the main characteristic volatile compounds in banana peel were esters, which were consistent with previous studies [36]. Most of the important volatile compounds were induced by melatonin treatment, especially on Day 4 (Additional file 7: Table S2). In addition, 16 genes related to volatile compound metabolic process were up-regulated after melatonin treatment (Additional file 9: Excel S1). Some esters were correlated to antibacterial function, such as texanol isobutyrate, isoamyl isobutanoate. In this study, higher content of these volatile compounds were found in the melatonin-treated banana peel after 4 days of storage, which was positively correlated with a higher expression level of genes for aromatic compound metabolic process (Fig. 8). A previous research on tomato also indicated that melatonin could alter the volatile compounds contents via regulating related gene expressions during tomato ripening process [7]. Therefore, we speculated that melatonin treatment could induce the release of volatile compound in banana peel, which may also be associated with improved banana defense against pathogen infection.

Fruit lipids are the major constituents of biological membranes that can sense extracellular conditions [37]. The main biological functions of lipids include storing energy and signaling [38]. In addition, wax and cutin, which are not constituents of the lipid bilayer, but are components derived from lipids, contribute to the reduction of tissue injury [37]. In this study, 32 genes were involved in ether lipid metabolism according to KEGG analysis, and 22 of them showed a higher expression level in the melatonin-treated banana compared to the control, including *cycloartenol-C-24-methyltransferase, fatty acid hydroxylase, gibberellin 2-beta-dioxygenase, L-ascorbate oxidase, phosphoinositide phospholipase C 4, sesquiterpene synthase 5* and others (Additional file 9: Excel S1). These features indicate that lipids metabolism might be induced by melatonin treatment to increase the ability of stress resistance in banana peel.

### Melatonin treatment could activate the chitin-mediated defense pathway and some pathogenesis-related proteins

Chitin is one of the major structural components of the cell walls of many pathogenic fungi. Therefore, plants always accumulate chitinases to degrading the fungi cell wall in the defense reaction against the fungal pathogen [39]. However, all the differentially expressed *chitinase* genes were down-regulated after melatonin treatment in this study (Additional file 9: Excel S1). In contrast, most genes related to response to chitin were significantly up-regulated by melatonin treatment (Additional file 9: Excel S1). It has been reported that loss of chitin responsive genes enhanced the disease susceptibility of *Arobidopsis* [40]. It suggested that melatonin treatment could activate the chitin-mediated defense pathway in banana peel, which is beneficial for enhancing resistance to banana anthracnose.

Pathogenesis-related proteins are proteins produced in plants upon pathogen attack and many proteins found in plants are pathogen-related proteins [41]. Those mainly include thaumatin-like proteins, chitinases, *β*-1,3-glucanase, proteinase-inhibitor, endoproteinase, peroxidase, lipid-transfer protein, defensin, and others [41]. In this study, only a few of them were up-regulated significantly after melatonin treatment, including four *peroxidases* and two *lipid-transfer proteins* (Additional file 9: Excel S1). Since PR proteins are induced as part of systemic acquired resistance (SAR), our result showed that melatonin treatment might not completely activate SAR of banana fruit.

## Conclusion

The results of this study demonstrated that exogenous melatonin treatment significantly delayed banana fruit senescence and anthracnose incidence, not by killing pathogens on the fruit surface. After melatonin treatment, receptor protein kinases sent the signal to auxin, ethylene, ROS and MAPK (Fig. 9). The synthetic genes of fruit physical characters were up-regulated, and the contents of most substances were increased significantly, including cell wall, waxes, lipids and volatile components. The fruit senescence was delayed significantly, and the invasion of anthracnose was largely blocked on banana fruit (Fig. 9). Although PR proteins were rarely activated in the melatonin-treated samples, a great number of stress response related proteins and secondary metabolites were induced by melatonin treatment (Fig. 9), which might play a vital role in response to anthracnose infection.

## Methods

### Plant materials and treatment

Yellow banana (*Musa acuminate* L. AAA group, cv. Brazilian) fruit were bought from a commercial orchard in Guangzhou, China. Fruit fingers with uniform shape, color, size and little mechanical injury were selected and dipped for 3 min in distilled water (control), or 10 mM melatonin (Aladdin, USA). Then the fruit from both treatments were stored at 25 °C with 85–90% relative humidity, and sampled at 1 and 4 days after treatment. Each treatment consisted of five biological replicates, with 12 fruit fingers for each replicate. Peel tissues were taken from the middle part of each finger and ground into powder with liquid nitrogen and stored at -80 °C for further analysis.

### Determination of physiological parameters of banana fruit and *Colletotrichum musae*

Fruit color was measured as described by our previous research [42], and color changes were quantified as the hue angle with the formula *h* = 180° + tan^− 1^ (b*/a*). Fruit firmness was measured with a penetrometer GY-1 (Hangzhou Scientific Instruments) as described by Huang et al. [43]. *Colletotrichum musae* was isolated and purified from banana fruit peel using PDA medium. The disease spot diameter was measured at 4 and 6 days after melatonin treatment at four concentrations (0, 0.05, 0.1 and 0.5 mM).

### RNA extraction and RNA-seq (quantification) analysis

Total RNA was extracted from banana peel according to Li et al. [44], and DNA contamination was first removed by DNase I treatment. Then mRNA was enriched by oligo (dT) magnetic beads and fragmented into short fragments. First strand cDNA was synthesized using random hexamer primer, and then buffer, dNTPs, RNase H and DNA polymerase I were added to synthesize the second strand cDNA. The double strand cDNA was further purified with magnetic beads. End reparation and 3′ adenine addition was then performed. Finally, sequencing adaptors were ligated to the fragments before the fragments, which were enriched by PCR amplification. During the QC step, Agilent 2100 Bioanaylzer and ABI StepOnePlus Real-Time PCR System were used to qualify and quantify of the sample library. The library products were ready for sequencing via Illumina HiSeq™ 2000. Three biological replicates were used for RNASeq. Clean reads were obtained by removing reads containing adapters, reads containing ploy-N, and low-quality reads from raw data. The read length is 49 basepare. After data quality statistics, clean reads were mapped to banana genome sequences (https://banana-genome-hub.southgreen.fr/) by *BWA* software [45]. By mapping reads on the reference genome to statistical distribution reads on each chromosome, could help determine the reads coverage on chromosome, and the number of gene distribution. Gene expression level was quantified by RSEM software package. FPKM method is used in calculated expression level. Differentially expressed genes (DEGs) screening was performed based on Noiseq package method [46]. Noiseq method can screen differentially expressed genes between two groups containing biological replicates. Differentially expressed genes were screened according to the following criteria: Foldchange ≥2 and diverge probability ≥0.8. Finally, WEGO software [47] and KEGG [48] were used for biological functional classification analysis for the differentially expressed genes.

### Real-time quantitative PCR validation of RNA-seq data

Specific primers for q-PCR were shown in Additional file 8: Table S3 which were designed using the Primer Premier 6.0. Actin was used to normalize the content of cDNA, q-PCR was performed as described by Li et al. [13]. and repeated four times.

### Analyses of volatile compounds using gas chromatography coupled to mass spectrometry (GC-MS)

Two grams of each sample were grounded and homogenized with 4 mL NaCl saturated solution. Then the sample was held for 15 min at 40 °C before collecting volatile compounds. Volatile compounds were collected for 45 min followed by the method described by Jing et al. [49]. GC-MS analysis was performed according to the method of Jing et al. [49] using a GC-2010 gas chromatography (Shimadzu, Suzhou, China) equipped with a GC MS-QP2010 plus mass spectrometer (Shimadzu, Suzhou, China). Volatile compounds separation was conducted using a 30 m Rxi-5MS capillary column (0.25 mm i.d.) with split/splitless injector. Samples (1 μL) were injected into the injector and the analysis was repeated three times. The quantity of each compound was determined by the ratio of the peak area of a particular component to the peak area of cyclohexanone that was used as an internal control.

### Determination of IAA content and cell wall components in banana peel after melatonin treatment

IAA content was determined according to the manufacturer’s instructions. Briefly, 0.1 g fruit peel was suspended in 1 ml cold reagent I for 12 h. After 8000 g centrifugation, the supernatant was collected and evaporated, and then the sediment was eluted three times with 0.5 mL solution II. The lower aqueous phase was extracted twice with ethyl acetate, and then was evaporated and dissolved in solution III for high performance liquid chromatography (HPLC) analysis (Rigol L3000). The contents of cell wall components were determined according to the method of Zhao et al. [50], including cellulose, hemicellulose, WSP, ISP, CSP and lignin.

### Statistical analysis

Physiological parameters were measured and differentially expressed genes (transcriptome quantification) were analyzed using at least three biological replications. The data presented here are averages of three biological replicates. The false discovery rate method was used to determine differential gene expression using ‘FDR ≤0.001 and an absolute value of log_2_Ratio ≥1’ as the threshold to judge the significance of gene expression difference. Differences between different samples were statistically analyzed using Student’s t-test (*P* < 0.05). PLS-DA and OPLS-DA were also performed in volatile compounds analysis. The volatile compounds, with predictive VIP > 1 (OPLS-DA) and *p* < 0.05 (Student’s t-test), were recognized as important metabolites.

## Additional files

Additional file 1: **Figure S1.** Go functional classification of differentially expressed genes after melatonin treatment. (DOCX 363 kb)

Additional file 2: **Figure S2.** Cluster of the signal transduction related genes using ClueGo in Cytoscape. (DOCX 139 kb)

Additional file 3: **Figure S3.** Cluster of the transcription factor related genes using BinGo in Cytoscape. (DOCX 1516 kb)

Additional file 4: **Figure S4**. Schematic of the ‘response to stress’ using the MapMan visualization platform. (DOCX 510 kb)

Additional file 5: **Figure S5.** GC profiles of the volatile compounds from banana peel. (DOCX 51 kb)

Additional file 6: **Table S1**. Alignment statistics result with banana genome for all samples. (DOCX 17 kb)

Additional file 7: **Table S2.** Volatile components in banana peel. (DOCX 24 kb)

Additional file 8: **Table S3.** The primers used for q-PCR. (DOCX 25 kb)

Additional file 9: **Excel S1.** Differentially expressed genes in banana peel after melatonin treatment. (XLSX 78 kb)

## Data Availability

Raw data of RNA-seq has been uploaded to NCBI Sequence Read Archive (SRA), submission: SUB5221938.

## Abbreviations

CSP: Covalent-soluble pectin
DGE: Digital gene expression profile
GC-MS: gas chromatography coupled to mass spectrometry.
HPLC: High performance liquid chromatography
ISP: Ionic-soluble pectin
MAPK: Mitogen-activated protein kinase
NCS1: Six S-norcoclaurine synthases
OPLS-DA: Orthogonal projection to latent structures discriminant analysis
PLS-DA: Projection to latent structures discriminant analysis
q-PCR: Real-time quantitative PCR
RLKs: Receptor-like kinases
ROS: Reactive oxygen species
SAR: Systemic acquired resistance
WSP: WAter-soluble pectin
