# Supplementary material for: Comparative transcriptomic and metabolic analysis reveals the effect of melatonin on delaying anthracnose incidence upon postharvest banana fruit peel
Source: BMC Plant Biol. 2019 Jul 1;19:289. doi: 10.1186/s12870-019-1855-2 (PMC6604187; doi:10.1186/s12870-019-1855-2)

Additional file 3 Figure S3. Cluster of the transcription factor related genes using BinGo in Cytoscape.


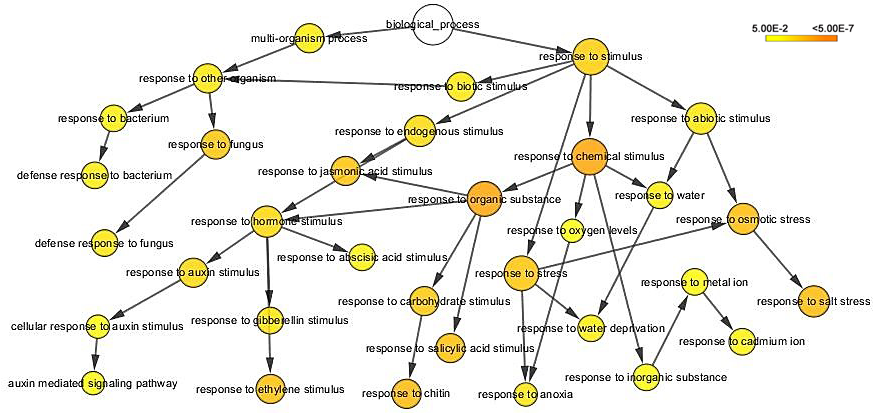

Supplement: Supplementary file 3 — Figure S3. Cluster of the transcription factor related genes using BinGo in Cytoscape. (DOCX 1516 kb) [file 12870_2019_1855_MOESM3_ESM.docx]
