# Supplementary material for: Comparative transcriptomic and metabolic analysis reveals the effect of melatonin on delaying anthracnose incidence upon postharvest banana fruit peel
Source: BMC Plant Biol. 2019 Jul 1;19:289. doi: 10.1186/s12870-019-1855-2 (PMC6604187; doi:10.1186/s12870-019-1855-2)

Additional file 4 Figure S4. Schematic of the ‘response to stress’ using the MapMan visualization platform. The analysis of homologous sequence showed that 580 differentially expressed banana genes could be matched to 334 Arabidopsis genes. MapMan (3.6.0RC1) analysis was performed by using Arabidopsis gene ID. The logarithm of gene expression ratios (melatonin treatment/control) base 2 were used in MapMan analysis. The red or green squares indicate the up or down-regulated genes involved in corresponding metabolism.


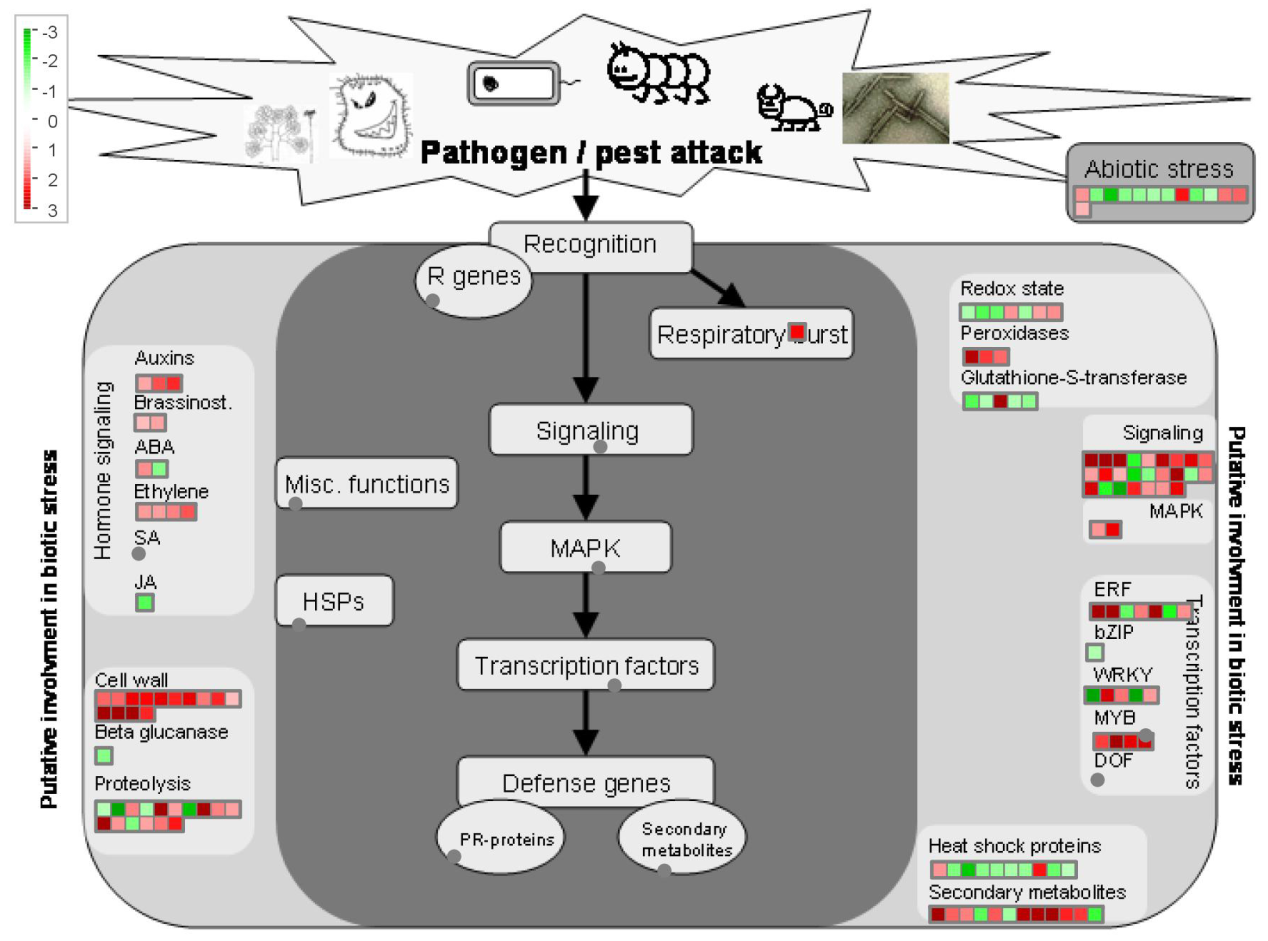

Supplement: Supplementary file 4 — Figure S4. Schematic of the ‘response to stress’ using the MapMan visualization platform. (DOCX 510 kb) [file 12870_2019_1855_MOESM4_ESM.docx]
