# Supplementary figures and images for: Comparative transcriptomic and metabolic analysis reveals the effect of melatonin on delaying anthracnose incidence upon postharvest banana fruit peel
Source: BMC Plant Biol. 2019 Jul 1;19:289. doi: 10.1186/s12870-019-1855-2 (PMC6604187; doi:10.1186/s12870-019-1855-2)

Additional file 5 Figure S5. GC profiles of the aromatic compounds from banana pericarps.


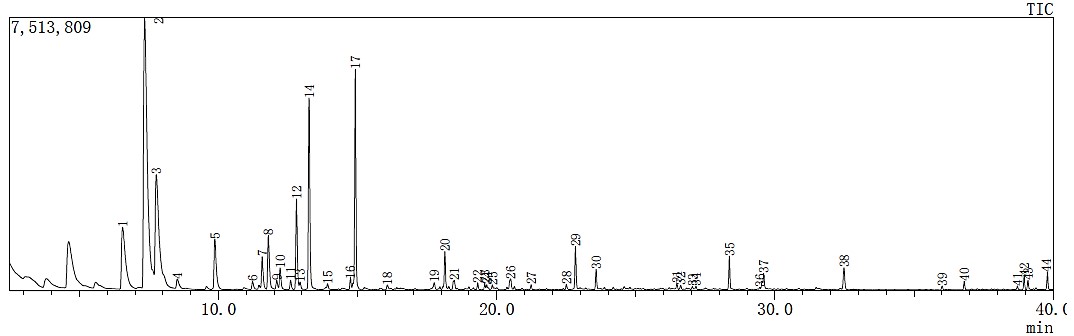

Supplement: Supplementary file 5 — Figure S5. GC profiles of the volatile compounds from banana peel. (DOCX 51 kb) [file 12870_2019_1855_MOESM5_ESM.docx]
