# Supplementary material for: Comparative transcriptomic and metabolic analysis reveals the effect of melatonin on delaying anthracnose incidence upon postharvest banana fruit peel
Source: BMC Plant Biol. 2019 Jul 1;19:289. doi: 10.1186/s12870-019-1855-2 (PMC6604187; doi:10.1186/s12870-019-1855-2)
Supplement: Supplementary file 6 — Table S1. Alignment statistics result with banana genome for all samples. (DOCX 17 kb) [file 12870_2019_1855_MOESM6_ESM.docx]

Additional file 6: Table S1. Alignment statistics result with banana genome for all samples.

| Sample Name | Clean reads | Total Mapped Reads | Genome map Rate | Unique Match | Multi-position Match | Gene map Rate | Expressed Genes |
| --- | --- | --- | --- | --- | --- | --- | --- |
| Control-1 | 12137883 | 9875648 | 81.36% | 9391212 | 484436 | 78.63% | 26721 |
| Control-2 | 12134027 | 9869496 | 81.34% | 9355933 | 513563 | 78.68% | 26347 |
| Control-3 | 12122542 | 9877247 | 81.48% | 9076903 | 800344 | 77.18% | 26034 |
| Melatonin-1 | 12140399 | 9936708 | 81.85% | 9425417 | 511291 | 78.16% | 26452 |
| Melatonin-2 | 12132994 | 9905029 | 81.64% | 9409771 | 495258 | 78.60% | 26282 |
| Melatonin-3 | 12140071 | 9901893 | 81.56% | 9404361 | 497532 | 78.59% | 26384 |
