# Supplementary material for: Comparative transcriptomic and metabolic analysis reveals the effect of melatonin on delaying anthracnose incidence upon postharvest banana fruit peel
Source: BMC Plant Biol. 2019 Jul 1;19:289. doi: 10.1186/s12870-019-1855-2 (PMC6604187; doi:10.1186/s12870-019-1855-2)
Supplement: Supplementary file 7 — Table S2. Volatile components in banana peel. (DOCX 24 kb) [file 12870_2019_1855_MOESM7_ESM.docx]

Additional file 7: Table S2. Volatile components in banana pericarp.

| **No.** | **Aromatic compounds** |  | **Ratio** |  |  | **Quality** | **RT (min)** | **RI** | |  |
| --- | --- | --- | --- | --- | --- | --- | --- | --- | --- | --- |
|  |  | **C4/C1** | **T4/C1** | **T1/C1** | **T4/C4** |  |  |  |  |  |
| **Esters** | | | | | | | | |  |  |
| 1 | 2-Acetoxypentane | 0.459 | 0.798 | 1.015 | 1.737 | 97 | 6.554 | 820 | |  |
| 2 | Isopentyl acetate | 0.641 | 1.447 | 1.268 | 2.257 | 97 | 7.348 | 820 | |  |
| 4 | Isobutyl isobutyrate | 0.774 | 1.547 | 1.165 | 2.000 | 97 | 8.520 | 856 | |  |
| 5 | Isobutyl butyrate | 0.616 | 1.058 | 0.112 | 1.718 | 99 | 9.867 | 920 | |  |
| 6 | n-Butyl butanoate | 0.896 | 1.067 | 13.242 | 1.191 | 97 | 11.221 | 984 | |  |
| 7 | Isobutyl isovalerate | 0.576 | 1.338 | 0.134 | 2.323 | 97 | 11.571 | 955 | |  |
| 8 | isoamyl isobutanoate | 0.719 | 1.726 | 0.819 | 2.401 | 95 | 11.793 | 955 | |  |
| 9 | 4-Hexenyl acetate | 1.674 | 3.098 | 27.054 | 1.850 | 96 | 12.090 | 992 | |  |
| 10 | 1-Methylbutylbutanoate | 0.532 | 0.693 | 0.956 | 1.304 | 97 | 12.213 | 1019 | |  |
| 11 | 3-Heptenyl acetate | 0.607 | 0.933 | 0.932 | 1.538 | 85 | 12.592 | 986 | |  |
| 12 | 2-Methylheptyl acetate | 0.497 | 0.829 | 1.063 | 1.667 | 88 | 12.802 | 1118 | |  |
| 13 | Butyl isovalerate | 0.699 | 0.980 | 1.184 | 1.400 | 96 | 12.930 | 1019 | |  |
| 14 | Isoamyl butanoate | 0.726 | 1.327 | 1.265 | 1.827 | 96 | 13.257 | 1019 | |  |
| 15 | 1-Methylbutyl pentanoate | 0.479 | 0.814 | 0.944 | 1.697 | 90 | 13.922 | 1118 | |  |
| 16 | 3-Methylbutyl-2-methylbutanoat | 1.004 | 1.962 | 1.042 | 1.955 | 95 | 14.733 | 1054 | |  |
| 17 | Isoamyl isovalerate | 0.652 | 1.488 | 1.157 | 2.282 | 98 | 14.919 | 1054 | |  |
| 20 | Isopentyl isobutyrate | 0.619 | 1.134 | 0.700 | 1.833 | 84 | 18.133 | 955 | |  |
| 22 | n-hexylisopentanoate | 0.841 | 1.184 | 0.488 | 1.408 | 96 | 19.312 | 1218 | |  |
| 23 | Isopentylhexanoat | 2.690 | 3.323 | 1.622 | 1.236 | 95 | 19.568 | 1218 | |  |
| 24 | 4-Hexen-1-yl valerate | 1.149 | 2.753 | 1.336 | 2.396 | 94 | 19.638 | 1290 | |  |
| 25 | Ethyl 3-oxoheptanoate | 1.006 | 1.490 | 0.927 | 1.482 | 79 | 19.843 | 1219 | |  |
| 26 | 1,1-Dipropoxyheptane | 0.952 | 1.742 | 0.970 | 1.830 | 80 | 20.496 | 1401 | |  |
| 27 | 1,3,5-Tri-O-acetyl-2,4-dideoxy-2,4-dimethylpentitol | 0.608 | 1.255 | 0.856 | 2.064 | 85 | 21.237 | 1623 | |  |
| 34 | Ethyl isobutyrylacetate | 4.108 | 6.140 | 0.286 | 1.495 | 78 | 27.158 | 1056 | |  |
| 36 | Texanol isobutyrate | 0.131 | 0.319 | 0.341 | 2.446 | 86 | 29.450 | 1605 | |  |
| 38 | Cyclohexylmethyl tridecyl oxalate | 0.384 | 0.660 | 0.598 | 1.718 | 77 | 32.483 | 2606 | |  |
| 40 | Diisobutyl-phthalat | 0.012 | 0.028 | 0.110 | 2.337 | 97 | 36.800 | 1908 | |  |
| 41 | Dibutyl phthalate | 0.012 | 0.020 | 0.093 | 1.736 | 97 | 38.713 | 2037 | |  |
| **Phenylpropanoid** | | | | | | | | | | |
| 29 | Eugenol | 0.712 | 1.202 | 0.858 | 1.689 | 97 | 22.829 | 1392 | |  |
| 33 | 2,6-Dimethoxy-4-allylphenol | 0.617 | 0.789 | 0.863 | 1.280 | 83 | 27.029 | 1581 | |  |
| 35 | Elemicin | 0.564 | 0.844 | 1.126 | 1.496 | 85 | 28.359 | 1550 | |  |
| 37 | Methoxyeugenol | 0.526 | 0.526 | 1.134 | 0.999 | 85 | 29.590 | 1581 | |  |
| **Others** | | | | | | | | | | |
| 19 | 4-Ethyl-1-hexyn-3-ol | 0.688 | 1.265 | 0.967 | 1.840 | 80 | 17.754 | 912 | |  |
| 21 | 2-Methyl-5-nitro-3-hexanol | 0.692 | 0.975 | 0.750 | 1.408 | 82 | 18.472 | 1133 | |  |
| 30 | Amyloxide | 1.085 | 1.744 | 0.809 | 1.607 | 87 | 23.570 | 1091 | |  |
| 31 | Humulen-(v1) | 4.339 | 3.099 | 11.547 | 0.714 | 91 | 26.475 | 1494 | |  |
| 39 | Octadeamethyl-cyclononasiloxane | 0.078 | 0.082 | 0.219 | 1.053 | 80 | 36.007 | 1860 | |  |

RT: Retention time; RI: Retention index. C4: Content of aromatic compounds components for control fruit at day 4; C1: Content of aromatic compounds for control fruit at day 1; T4: Content of aromatic compounds for melatonin-treated fruit at day 4; T1: Content of aromatic compounds for melatonin-treated fruit at day 1. The ratio represents the significant difference (*P* < 0.05) among different samples. No. indicates the peak numbers which were the same as shown in Additional file Figure S1
