# Supplementary material for: Comparative transcriptomic and metabolic analysis reveals the effect of melatonin on delaying anthracnose incidence upon postharvest banana fruit peel
Source: BMC Plant Biol. 2019 Jul 1;19:289. doi: 10.1186/s12870-019-1855-2 (PMC6604187; doi:10.1186/s12870-019-1855-2)
Supplement: Supplementary file 8 — Table S3. The primers used for q-PCR. (DOCX 25 kb) [file 12870_2019_1855_MOESM8_ESM.docx]

Additional file 8: Table S3. The primers used for q-PCR.

| Gene ID | Description | Sequence Length | Product Length | Primer sequence | | Tm |
| --- | --- | --- | --- | --- | --- | --- |
| GSMUA_Achr6T14620_001 | Putative Peroxidase 52 | 795 | 128 | For: | GCACTCCGACCAGGAGCTGTTCAA | 63.4 |
|  |  |  |  | Rev: | AGAGGCTAATGTTGCCCATCTTCACCAT | 63.3 |
| GSMUA_Achr10T14220_001 | Peroxidase 4 | 870 | 299 | For: | TCCTGGACGACACACCGACGAT | 61.7 |
|  |  |  |  | Rev: | TGCGAACTTACTGATGAGACTGCTGAG | 61.7 |
| GSMUA_Achr7T18420_001 | 1-aminocyclopropane-1-carboxylate oxidase | 957 | 117 | For: | GTGGCTCAGACTGATGGCAACAG | 60.1 |
|  |  |  |  | Rev: | CTCCTTCTTCTCCTCCGCTTCCTT | 60.2 |
| GSMUA_Achr6T00870_001 | 1-aminocyclopropane-1-carboxylate oxidase | 882 | 199 | For: | AGGTCGTGAGCAATGGCGTCTA | 60.1 |
|  |  |  |  | Rev: | TTGTCTGAGAACTTGGTCTTGGTGTAG | 59.7 |
| GSMUA_Achr4T11940_001 | Luminal-binding protein 4 | 2169 | 172 | For: | TCTTGAGCGGAGAAGGTGGTGAA | 60 |
|  |  |  |  | Rev: | GTCTGCTGATCTTGGTAAGTCGTGAA | 59.8 |
| GSMUA_Achr3T27250_001 | Luminal-binding protein 5 | 1806 | 210 | For: | AAGGATTCCGAAGGTCCAACAACTCT | 60.9 |
|  |  |  |  | Rev: | GTCATCACTCCACCAACCGTCTCA | 60.9 |
| GSMUA_Achr11T04480_001 | S-norcoclaurine synthase 1 | 1071 | 222 | For: | TGAACGACGTTGAAGGACTCCATATCA | 61 |
|  |  |  |  | Rev: | GAGGACCAACCACCGCCGAATA | 60.6 |
| GSMUA_Achr11T04520_001 | Putative S-norcoclaurine synthase 1 | 1068 | 100 | For: | CCCGCTCAACCTCTCACTTTCAGA | 60.9 |
|  |  |  |  | Rev: | CCAGATTCGTCGCCATCACCTCT | 60.8 |
| GSMUA_Achr11T04500_001 | S-norcoclaurine synthase 1 | 1068 | 189 | For: | CGCTCTCATCGCTAACATCGGTGATA | 61.5 |
|  |  |  |  | Rev: | ACATACTTCGGCTTGCACTCCTTCA | 61.1 |
| GSMUA_Achr7T26580_001 | UDP-glucose 6-dehydrogenase | 393 | 114 | For: | TAGCATCTACGATCCTCAAGTGACAGTG | 61.1 |
|  |  |  |  | Rev: | GCATAATCCAGCTTCCTGAACTCATCC | 60.7 |
| GSMUA_Achr1T17550_001 | Catalase isozyme 2 | 339 | 176 | For: | CTGGCAAGCGTGAGAAGAATGTGATT | 61 |
|  |  |  |  | Rev: | CGACAGGAACGAGATCCAGATGCT | 60.8 |
| GSMUA_Achr11T03210_001 | Putative Ethylene-responsive transcription factor ERF024 | 519 | 125 | For: | GCCGCAACTGCTGGTGAACA | 59.8 |
|  |  |  |  | Rev: | ACGGATAGCTCCACCTCCACAG | 59.5 |
| GSMUA_Achr11T15810_001 | Ethylene-responsive transcription factor ERF071 | 600 | 191 | For: | AAGCAAGTCTCCGTCGCTCTCG | 60.9 |
|  |  |  |  | Rev: | ATCCCAATATTCCCTGTCACAACCTCAT | 61 |
| GSMUA_Achr1T23990_001 | putative MYB DNA-binding domain superfamily protein [Zea mays] | 993 | 264 | For: | ATCTGGACCTGCTCCGCCTCTT | 61.4 |
|  |  |  |  | Rev: | AGATACAAGTAGCTGTGCCACAACCT | 60.9 |
| GSMUA_Achr1T10080_001 | Putative Probable WRKY transcription factor 40 | 1008 | 135 | For: | GCACCACCAGCGACGATTCTTG | 60.7 |
|  |  |  |  | Rev: | GCCCATATTTCCTCCATTGATAGCCATC | 60.9 |
| GSMUA_Achr6T05880_001 | Putative WRKY transcription factor 6 | 1431 | 220 | For: | GGTCCTCGTGCCAAGACAGTTCA | 61.1 |
|  |  |  |  | Rev: | TTGCTCGGTGCTACTCGCTTCC | 61.2 |
| GSMUA_Achr6T33100_001 | Mitogen-activated protein kinase 5 | 1104 | 260 | For: | TTACGGCATCGTCTGTTCGGTGAT | 61 |
|  |  |  |  | Rev: | CTTGGTTGGAGCGGATTATATGGTGAAG | 61 |
| GSMUA_Achr8T27900_001 | Putative Acidic endochitinase SE2 | 654 | 150 | For: | ACGTGTGGGTGCAGTTCTACAACAA | 61.7 |
|  |  |  |  | Rev: | GCCGTAGTTGGACGCAGTCTTGAT | 61.7 |
| GSMUA_Achr9T29630_001 | Putative E3 ubiquitin-protein ligase PUB23 | 1857 | 222 | For: | AACTCTTCTCCGTCCCTCACTTCTC | 60.2 |
|  |  |  |  | Rev: | CCACCTCTTCGCCTCTTCCTCTT | 60.1 |
| GSMUA_Achr7T03450_001 | Polyphenol oxidase, chloroplastic | 1848 | 269 | For: | ACGACGAGAACGCCGACTTAGT | 60.1 |
|  |  |  |  | Rev: | TATCTCCTCCTCATCTTCCTTCTCCTTG | 60 |
| GSMUA_Achr10T03730_001 | Actin | 1134 | 66 | For: | TGGTATGGAAGCCGCTGGTA | 60.2 |
|  |  |  |  | Rev: | TCTGCTGGAATGTGCTGAGG | 60.4 |
